# Selective Production of Diesel-Range Hydrocarbons from Catalytic Pyrolysis of Polypropylene Waste Using Modified Natural Zeolites: Interplay of Acidity, Temperature, and Reaction Parameters

**DOI:** 10.3390/polym18101147

**Published:** 2026-05-07

**Authors:** Joaquín Hernández-Fernández, Rafael González-Cuello, Rodrigo Ortega-Toro

**Affiliations:** 1 Chemistry Program, Department of Natural and Exact Sciences, San Pablo Campus, University of Cartagena, Cartagena de Indias 130015, Colombia; 2 Department of Natural and Exact Science, Universidad de la Costa, Barranquilla 080002, Colombia; 3 Food Packaging and Shelf-Life Research Group (FP&SL), Food Engineering Program, University of Cartagena, Cartagena de Indias 130015, Colombia; rgonzalezc1@unicartagena.edu.co (R.G.-C.); rortegap1@unicartagena.edu.co (R.O.-T.)

**Keywords:** catalytic pyrolysis polypropylene, natural zeolites, diesel-range hydrocarbons, plastic waste valorization, circular economy, ANOVA, correlation analysis

## Abstract

In the context of this study, it is investigated whether catalytic pyrolysis of post-consumer polypropylene might prove an interesting route to the generation of liquid hydrocarbon materials from plastic waste. The optimum product selectivity can be achieved using the appropriate catalyst. To address this problem, we tested three altered natural zeolites as follows: H-ZN, AT-ZN, and AA-ZN, according to a factorial design which accounts for temperature (400–500 °C), heating rate (10–20 °C per minute), and catalyst loading (5–10 percent by weight). Initially, we verified by thermogravimetric and micro-Raman analyses the thermal behavior of the catalytic systems and the consistency of the polypropylene feedstock. This work confirms that the catalyst assists in initiating the chain-scission process, as changes to the zeolites are responsible for the breakdown of polypropylene at a lower temperature. H-ZN showed high liquid recovery (75.4 wt%), particularly under moderate conditions, as confirmed by product-yield analysis. On the other hand, AT-ZN was more conducive to gas formation and light-fraction production at higher temperatures. H-ZN kept the diesel-range fraction (C12–C20) stable nearly to 51%, according to GC–MS; AT-ZN shifted selectivity to gasoline-range hydrocarbons (C6–C11), up to 57% under severe conditions. AA-ZN showed intermediate behavior. The overall conversion and molecular profile of the liquid products were influenced not only by catalyst acidity, temperature, and their interactions but also by Pearson correlation and ANOVA. The results described above indicate that H-ZN is the most promising catalyst for selective polypropylene-to-diesel conversion and prove that modified natural zeolites are an inexpensive and scalable method for valorizing plastic waste in a circular economy.

## 1. Introduction

**Scheme 1:**
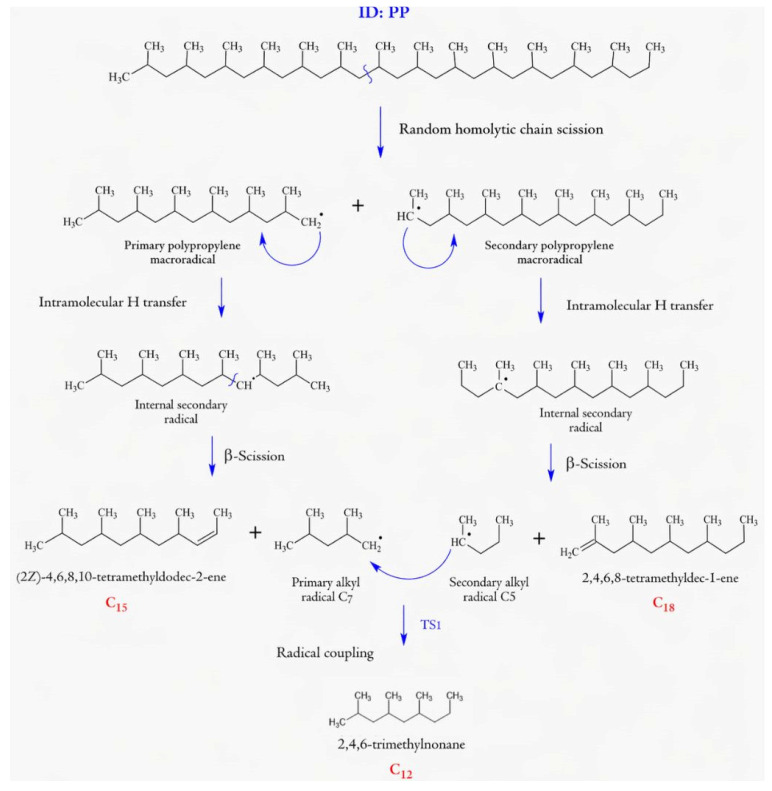
Simplified mechanistic representation of carbon–carbon bond cleavage during polypropylene pyrolysis, including random homolytic chain scission, intramolecular hydrogen transfer, β-scission, and the formation of representative olefinic and alkyl-radical fragments.

Plastic waste has become one of the most pressing environmental challenges of this century. Among commodity polymers, polypropylene (PP) is one of the most widely used materials in packaging, automotive components, and textile fibers [1,2,3]. Although mechanical recycling remains the most common strategy for managing PP waste, it has important limitations. The polymer undergoes progressive degradation after each recycling cycle, complicating the processing of contaminated waste streams and leading to deterioration of the original material properties [4,5]. In this context, catalytic pyrolysis has emerged as a promising alternative within the circular economy framework, since it enables the conversion of plastic waste into liquid hydrocarbons and other value-added products with fuel-like properties [6,7,8]. Unlike conventional thermal pyrolysis, which for PP generally requires temperatures above 425 °C and often leads to a high proportion of heavy waxes, catalytic pyrolysis can lower the apparent energy demand of the process and improve selectivity toward more valuable liquid fractions. From a mechanistic perspective, the thermal pyrolysis of polypropylene is generally described as a free-radical process initiated by random C–C bond scission, followed by β-scission, hydrogen transfer, and secondary reactions that generate olefins, paraffins, and heavier wax-like intermediates. In catalytic systems, particularly those involving acidic zeolites, the reaction network may additionally include acid-site-mediated cracking pathways, in which Brønsted acidity facilitates the formation of reactive intermediates and promotes further fragmentation, isomerization, cyclization, and aromatization of polymer-derived species. Therefore, catalyst acidity, pore architecture, and active-site accessibility are key factors that control the extent of C–C bond cleavage and the final distribution of hydrocarbon products. A simplified representation of the main bond-cleavage events commonly associated with polypropylene pyrolysis is shown in Scheme 1. This scheme illustrates the generally accepted sequence of random homolytic chain scission, intramolecular hydrogen transfer, and subsequent β-scission steps that lead to the formation of olefinic fragments and alkyl radicals. Although the present study does not aim to elucidate the reaction mechanism at the molecular level, this mechanistic framework is useful for rationalizing how catalyst acidity may influence product redistribution and hydrocarbon selectivity during polypropylene cracking.

Natural zeolites, such as clinoptilolite, have attracted considerable attention because of their low cost and wide availability, although chemical modification is usually required to enhance their surface acidity and porosity [9,10,11,12]. It has been reported that acid treatment, thermal activation, or conversion into the acid form can partially remove framework aluminum and exchange extra-framework cations, thereby increasing the number and strength of active sites involved in polypropylene cracking [13,14]. Despite advances in the use of synthetic zeolites, the performance of modified natural zeolites for directing product selectivity toward the diesel fraction (hydrocarbons in the C9–C25 range) still requires further optimization and systematic evaluation [15,16,17,18].

This study addresses that gap by systematically comparing the effects of three modification pathways of a natural zeolite, namely H-ZN, AT-ZN, and AA-ZN, on the product distribution obtained during the catalytic pyrolysis of post-consumer polypropylene. The main novelty of this work lies in the comparative evaluation of the thermal response and structural properties of the modified zeolites after dealumination, thermal activation, and acid-form generation [19]. In addition, the selectivity of each catalytic system toward different hydrocarbon fractions was determined by gas chromatography–mass spectrometry, with particular attention to diesel-range enrichment and product redistribution by carbon number [20]. Mass balance analysis was also used to identify the operating conditions that maximize liquid oil yield, which can exceed 60% under optimized catalytic conditions. This approach makes it possible to determine not only which catalyst favors greater liquid recovery, but also which one preferentially directs selectivity toward lighter or intermediate hydrocarbon fractions of higher energetic interest. Therefore, this study aimed to evaluate the effect of modified natural zeolites on the overall yield and chemical composition of hydrocarbons produced during the catalytic pyrolysis of polypropylene. Under this approach, the process is positioned as a sustainability-oriented strategy that can transform low-degradability plastic waste into a high-value liquid stream, thereby contributing to more profitable waste management practices aligned with the Sustainable Development Goals.

## 2. Materials and Methods

### 2.1. Raw Materials and Preparation

For this research, post-consumer polypropylene collected from household plastic waste was used. The material was cleaned to remove organic contaminants, dried at 60 °C, and mechanically ground to a uniform particle size of approximately 2–3 mm to ensure homogeneous heat transfer [21]. Natural clinoptilolite zeolite, used as the catalytic precursor, was purchased from Agorgarzon, Colombia, and selected for its abundance and low operating cost [9]. The chemical reagents used for the modifications, including hydrochloric acid (HCl), sulfuric acid (H_2_SO_4_), and ammonium chloride (NH_4_Cl, ≥99.995%, Merck/Supelco, Darmstadt, Germany), were of analytical grade.

### 2.2. Catalyst Preparation and Modification

Natural zeolite was subjected to three different modification routes to evaluate its selective cracking capacity. Thermal activation [6]: Natural zeolite was washed with deionized water to remove surface impurities and then calcined in a muffle furnace at 500 °C for 4 h. This process aims to open the pore channels by removing hydration water and residual organic matter. Acid activation: An acid leaching treatment was performed using an HCl solution (0.5–1.0 M) under constant stirring at 80 °C. This procedure induces a controlled partial dealumination of the structural framework, increasing pore volume and accessibility to active sites. Acid form: Prepared via ion exchange using a 1.0 M NH_4_Cl solution. The mixture was refluxed at 80 °C for 6 h to replace the original metal cations with ammonium ions (NH_4_^+^). Finally, the material was filtered, dried, and calcined at 550 °C to promote ammonium decomposition and generate the Brønsted acid sites necessary for the carbenium ion mechanism [22].

The physicochemical characteristics of the modified zeolites (see Table 1) demonstrate a systematic evolution depending on the application techniques. A similar evolution of catalytic efficiency on polypropylene pyrolysis was also witnessed. The rise in the Si/Al ratio from 4.7–4.9 in natural zeolite to 7.9–8.0 in H-ZN is consistent with increasing dealumination, particularly when acid treatment and ion exchange are employed. These structural changes decrease the density of the framework aluminum and increase the concentration and strength of acid sites, especially Brønsted sites. Such chemical conversion leads to dramatic changes in texture. BET surface area increases from 25 to 26 m^2^/g in natural zeolite to 133 to 134 m^2^/g in H-ZN, and pore volume increases from 0.08 to 0.09 to 0.28 to 0.32 cm^3^/g, indicating that acid treatment and protonation modulate the chemical structure and make pores more accessible, probably in part by removing extra-framework species and partially rearranging the framework. The average pore diameter increases from 4 to 5 nm to 8 to 9 nm, meaning the pores are more open. This is of great significance for the diffusion of large polymer-derived intermediates. Therefore, the most important factor in catalysis is the change in acidity. The total acidity rises from 0.16–0.18 mmol NH_3_ g^−1^ in natural zeolite to 0.71–0.72 mmol NH_3_ g^−1^ in H-ZN and a movement from mostly weak Lewis sites to strong Brønsted acid sites occurs. This change is important because Brønsted acidity initiates carbocation-driven cracking, thereby helping control chain breaking and stabilizing hydrocarbon fragments. H-ZN therefore improves efficiency, resulting in a larger liquid yield and higher selectivity for diesel-range hydrocarbons, as evidenced by product analysis. On the other hand, AT-ZN shows only slightly increased surface area and acidity with mainly Lewis acid sites. This implies that thermal activation alone does not form the strong acid sites that are essential for the selective catalytic cracking process; thus, the process is less controlled and results in more gas being generated. AA-ZN is in the middle: partial dealumination raises the surface area and acidity but does not reach the Brønsted acid dominance of H-ZN. It holds moderate catalytic ability, with liquid yields and selectivity between the two alternatives.

### 2.3. TGA

Thermogravimetric analysis (TGA) is performed using a TA Instruments Discovery 55 TGA thermobalance, which operates over the temperature range of 25–900 °C with a controlled heating rate of 20 °C/min. The carrier gas used during the analysis is nitrogen (N_2_) with a precisely regulated flow rate of 0.83 ± 0.005 mL/s. This fundamental parameter provides information on the thermal stability and degradation properties of the analyzed substances.

### 2.4. Micro-Raman

Raman measurements were performed using a DXRT™ Raman Microscope (Thermo Fisher Scientific, Waltham, MA, USA) with 532 nm excitation (green laser). The laser radiation was focused using 50× and 100× objectives (numerical apertures (NAs) of 0.50 and 0.90, respectively; NA defines how much light the lens can gather). The laser power and acquisition settings were optimized through preliminary tests within the reported ranges.

### 2.5. Experimental System and Reactor

The catalytic pyrolysis experiments were carried out in a stainless steel fixed-bed reactor under an inert atmosphere with a constant nitrogen (N_2_) flow of 50 mL/min to prevent oxidation reactions [23]. The experimental design followed a multifactorial matrix (based on the previously described ANOVA analysis) that included the following variables: pyrolysis temperatures of 400, 450, and 500 °C; heating rates of 10 and 20 °C/min; and catalyst loadings of 5% and 10% *w*/*w* relative to the polymer mass.

In each trial, approximately 20 g of the mixture (PP + catalyst) was loaded into the reactor. The volatile products generated were passed through a condensation system cooled with a water–ethylene glycol bath at −5 °C to recover the liquid fraction (pyrolysis oil). Non-condensable gases were collected in Tedlar bags for later analysis, while the solid residue (char and spent catalyst) was recovered directly from inside the reactor after cooling [24].

### 2.6. Product Quantification and Characterization: GC-MS Analysis of Oil

The yields of the products (liquid, solid, and gas) were determined using a gravimetric mass balance. The chemical composition of the liquid fraction was analyzed by gas chromatography coupled with mass spectrometry, using a high-resolution capillary column. The identified compounds were classified by carbon chain length into four strategic ranges: light gasoline (C6–C11), diesel fraction (C12–C20), light kerosene/waxes (C21–C28), and heavy fractions (C29–C40) [25].

The chemical composition of the pyrolytic oil was analyzed by gas chromatography–mass spectrometry using a Thermo Scientific TRACE 1610 GC system coupled to an Orbitrap Exploris GC mass spectrometer, both supplied by Thermo Fisher Scientific (Waltham, MA, USA). A capillary column (DB-5MS type, 30 m × 0.25 mm × 0.25 µm) was used with helium as the carrier gas. The reported compositions are semi-quantitative, based on peak area normalization. The oven temperature program was configured to start at 40 °C, maintained for 2 min, and then increased to 300 °C at 10 °C min^−1^ [26].

## 3. Results and Discussion

### 3.1. Thermal and Micro-Raman Characterization

For thermal prediction, we undertook thermogravimetric and micro-Raman analyses of the catalytic systems and structural consistency of the polypropylene feedstock. TGA was used to evaluate the influence of zeolite modification on polypropylene degradation and to investigate catalyst-induced shifts in the cracking window. The vibrational fingerprint of the material was confirmed by micro-Raman spectroscopy of multiple sampling points. Collectively, these approaches provide a strong physicochemical basis for estimating the catalytic behavior of modified zeolites and the compositional uniformity of the polymer before pyrolysis.

**Figure 1:**
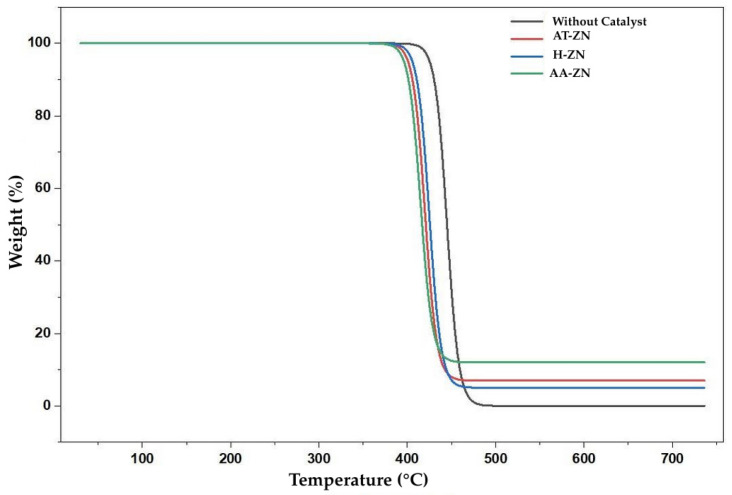
Thermogravimetric degradation curves of polypropylene under non-catalytic conditions and in the presence of modified natural zeolites (AT-ZN, H-ZN, and AA-ZN).

Thermogravimetric studies revealed that polypropylene reacts much more readily when modified natural zeolites are added. Figure 1 shows that the material is thermally stable in non-catalytic thermal pyrolysis, with degradation occurring at around 445–450 °C. When H-ZN, AT-ZN, and AA-ZN are involved, degradation starts at lower temperatures (meaning these catalysts can break the chains under milder conditions). AA-ZN has the most apparent influence, lowering the initiation of degradation by approximately 40–50 °C compared to the catalytic-free treatment. This corresponds to an obvious reduction in activation energy needed for cracking. Similar results have been obtained with natural zeolite systems in previous investigations, but the more significant change for AA-ZN is its increased catalytic effect on polypropylene conversion [27,28].

Since the conversion is effective at temperatures close to 400 °C, the data from the catalyst are relevant, as they fall within the 445–450 °C range typical of non-catalytic degradation. The energy savings from this nearly 10% operating temperature increase are direct, as lower temperatures reduce heat demand and may lower operating costs [29]. While the non-catalytic system leaves virtually no residues, the mass of the catalyzed sample residue ranges from 5 to 12%. This residue suggests both the presence of the solid catalyst and some carbonaceous material produced during catalytic cracking, rather than only incomplete conversion. AA-ZN (and AT-ZN to a lesser extent) will likely be superior due to catalyst modification, which makes acid sites more accessible, especially Brønsted acidity. This allows carbocation-mediated scission, resulting in faster breakdown of polypropylene. In terms of sustainability, this study reports that inexpensive modified natural zeolites could be considered an alternative to synthetic catalysts for converting polypropylene waste into useful products. In this way, process intensity is also reduced, and the environmental impact of plastic disposal is reduced [30,31,32].

**Figure 2:**
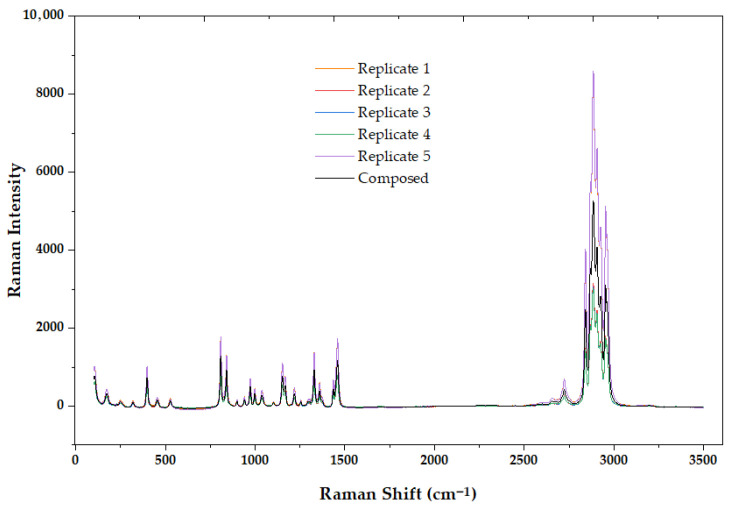
Micro-Raman spectra collected from five independent replicates of the polypropylene sample together with the composite spectrum in the 100–3500 cm^−1^ region.

The micro-Raman spectra (Figure 2) show the same vibrational fingerprint across all five replicates, while the composite spectrum closely reflects the sample’s overall spectral profile. The primary bands are all in the same Raman shift in each replicate, confirming that the chemical identity of the material does not change at multiple analysis sites. In fact, this sample shows no significant compositional heterogeneity, secondary phases, or microscale chemical alteration. The signature spectral differences between spectra are more in intensity levels, e.g., high-frequency at 2800−3000 cm^−1^ and the fingerprint at around 800−1500 cm^−1^, rather than the emergence of new bands or major peak shifts [33].

From a structural perspective, the strong cluster around 2800–3000 cm^−1^ correlates with C–H stretching vibrations characteristic of the aliphatic polymer backbone and methyl groups. The large signals at about 1450 cm^−1^ are due to the deformation modes of the CH_2_ and CH_3_ groups. The bands between 800 and 1200 cm^−1^ and 1300 and 1350 cm^−1^ correspond to skeletal vibrations and deformation patterns characteristic of polypropylene chains. Since all of these bands are present in all samples, the polymer matrix in these samples is chemically consistent. These changes in intensity are moderate but likely consistent with individual differences in focus, orientation, sampling volume, and/or surface texture. Replicate 5, where the greatest signal intensity was observed in some spectral areas, should not be interpreted as a sign of a chemically different domain but rather as a locally stronger Raman response. Overall, the Raman findings demonstrate that the studied polypropylene sample is spectroscopically reproducible and structurally homogeneous at the probed scale, reinforcing the representativeness of the feedstock used in later pyrolysis work [34,35,36].

### 3.2. Effect of Zeolite Type, Zeolite Quantity, Temperature, and Heating Rate on Thermal Pyrolysis Yields

**Figure 3:**
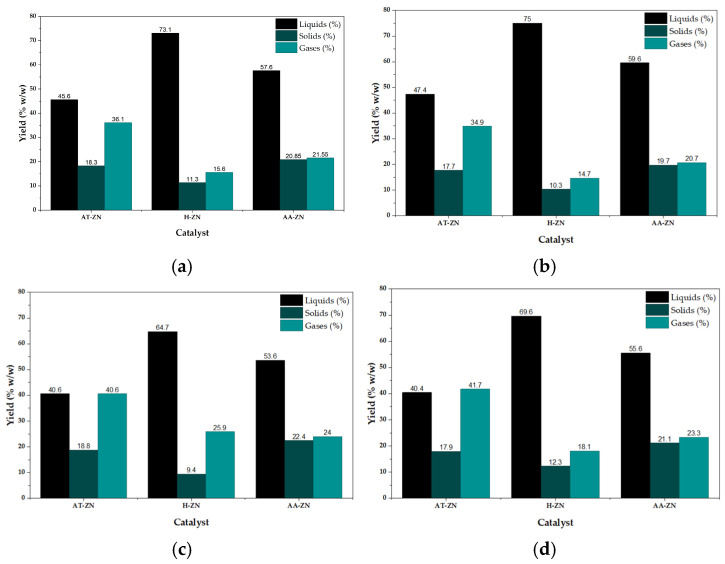
Product-yield distribution obtained at 400 °C during the catalytic pyrolysis of polypropylene over modified natural zeolites under different operating conditions: (**a**) 5 wt% catalyst and 10 °C min^−1^, (**b**) 5 wt% catalyst and 20 °C min^−1^, (**c**) 10 wt% catalyst and 10 °C min^−1^, and (**d**) 10 wt% catalyst and 20 °C min^−1^.

**Figure 4:**
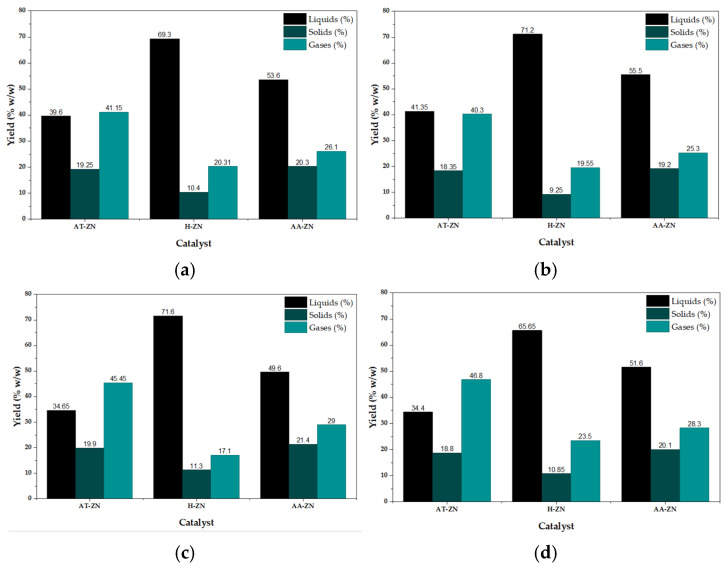
Product-yield distribution during the catalytic pyrolysis of polypropylene at 450 °C over modified natural zeolites under different operating conditions: (**a**) 5 wt% catalyst and 10 °C min^−1^, (**b**) 5 wt% catalyst and 20 °C min^−1^, (**c**) 10 wt% catalyst and 10 °C min^−1^, and (**d**) 10 wt% catalyst and 20 °C min^−1^.

**Figure 5:**
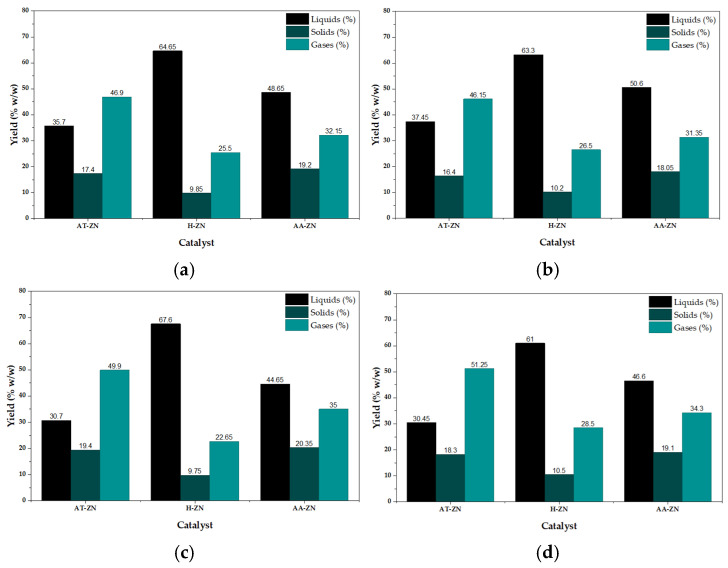
Product-yield distribution during the catalytic pyrolysis of polypropylene at 500 °C over modified natural zeolites under different operating conditions: (**a**) 5 wt% catalyst and 10 °C min^−1^, (**b**) 5 wt% catalyst and 20 °C min^−1^, (**c**) 10 wt% catalyst and 10 °C min^−1^, and (**d**) 10 wt% catalyst and 20 °C min^−1^.

To explore the effects of catalyst modification and operating conditions on the phase distribution of polypropylene pyrolysis products, we calculated gravimetric mass balances using a factorial design for the liquid, solid, and gaseous yields. Given that zeolite type, catalyst loading, reaction temperature, and heating rate all influence primary cracking, secondary vapor-phase reactions, and stable condensable intermediates, this group of parameters was chosen. This demonstrates that product selectivity in catalytic pyrolysis is influenced not only by temperature but also by the interaction among catalyst acidity, textural properties, and the thermal history of the polymer and volatile fragments. Trends in product yields at 400, 450, and 500 °C are shown in Figure 3, Figure 4 and Figure 5 and serve as a basis for comparing conditions to maximize liquid recovery while minimizing gas formation and solid residue.

Referring to Figure 3a–d, we can see that at 400 °C, the product distribution is greatly dependent on the nature of the modified zeolite used. H-ZN under all conditions yielded the highest liquid content, reaching up to 75.0 wt%. These results are in line with the literature, which suggests that the protonic zeolite exhibits the best catalytic potential for polypropylene cleavage under mild thermal conditions, due to its closer proximity to Brønsted acid sites, which could be utilized to cleave hydrocarbon chains. In contrast, AT-ZN shows worse performance, suggesting that simple thermal activation is less effective at generating acidic sites that drive the reaction into the liquid phase. H-ZN has low solid fractions during the range from Figure 3a–d, also showing that this does make the catalytic conversion more effective, since less unconverted material is encountered and residues that resemble coke are reduced.

The second central observation concerns the operating condition. Increasing the heating rate from 10 to 20 °C min^−1^ under steady catalyst loading had a modest effect on the liquid production, in particular for H-ZN and AA-ZN (Figure 3a,b and Figure 3c,d, respectively), and higher heating ramp also reduced the residence time of the primary vapor in the high-temperature area, which reduced secondary cracking, gasification and condensation of the liquid intermediates to lighter gases or solids. Instead, the addition of catalyst and the increase in the catalyst loading from 5 to 10 wt% did not increase liquid production, and a reduction in the liquid fraction was especially showed for H-ZN, which indicated an excessive amount of active sites or over-cracking causing more fractionation in primary condensable products to non-condensable gases (Figure 3a versus Figure 3c and Figure 3b versus Figure 3d). Overall, the optimum tradeoff between catalytic activity and product selectivity is realized by H-ZN at 400 °C with low catalyst loading and high heating rate (Figure 3a–d) [37,38].

From Figure 4a–d we observe that product distributions at 450 °C are more thermally demanding than at 400 °C, suggesting that the influence of temperature on cracking chemistry is greater [37]. Polypropylene deterioration at this elevated temperature is less caused by the initial generation of condensable intermediates and more by the evolution of such intermediates in the gas phase and on the catalyst surface [38]. H-ZN remains the most potent catalyst in all four subfigures to prepare liquid forms with a production yield between 65.65 and 71.6 wt%, thus evidencing that its acidic composition retains a strong capability to convert the polymer into condensable hydrocarbons. Nevertheless, the total liquid fraction is slightly lower than its maximum value at 400 °C, suggesting that some of the primary liquid products are consumed by later cracking steps [39].

At a temperature of 400–450 °C, the secondary scission reactions, that is, the β-scission of radical fragments and further fragmentation of oligomeric species that are already formed, become more significant. This is why the gas fraction increases compared to lower temperatures; in particular, AT-ZN has a gaseous fraction of 40.3–46.8 wt% [40]. These findings show that thermally activated zeolite has a limited capacity at high temperatures to stabilize intermediate hydrocarbon fragments prior to further fragmentation. At the same time, AA-ZN exhibits intermediate behavior, with liquid yields ranging between 49.6 and 55.5 wt%. This indicates that it is somewhat favorable for condensable products but does not demonstrate selectivity like H-ZN.

Another relevant feature of Figure 4a–d is that, at 450 °C, the effect of catalyst loading and heating rate becomes more sensitive to the catalyst nature. In H-ZN, increasing the catalyst amount does not lead to a systematic improvement in liquid yield; rather, under the combined condition of 10 wt% and 20 °C min^−1^ (Figure 4d), the liquid fraction decreases while the gas fraction rises. This behavior is consistent with a regime in which the higher availability of acid sites, together with the stronger thermal input, accelerates not only primary depolymerization but also the re-cracking of condensable vapors into lighter non-condensable molecules. Therefore, Figure 4a–d shows that 450 °C constitutes an intermediate mechanistic window: still favorable for liquid production, especially with H-ZN, but already sufficiently energetic to intensify secondary cracking and progressively shift the product slate toward gaseous compounds.

As shown in Figure 5a–d, at 500 °C, the reaction system clearly shifts toward a more severe cracking regime, in which the effect of temperature becomes dominant over the selectivity patterns observed at lower temperatures. Although H-ZN still provides the highest liquid yields among the three catalysts, its performance is lower than that observed at 400 and 450 °C, with liquid fractions ranging from 61.0 to 67.6 wt%. At this temperature, the optimum acidic catalyst does not entirely suppress the conversion to lighter non-condensable products from primary condensable hydrocarbons. AT-ZN has the lowest overall liquid yields (30.45–37.45 wt%) and also the highest gas output (up to 51.25 wt%) (shown in Figure 5d), while AA-ZN shows intermediate liquid yields between 44.65 and 50.6 wt%. Taken together, Figure 5a–d demonstrates the greater competition between the formation of liquid and the formation of gas with an increasing temperature up to 500 °C, which diminishes the capacity of the catalyst to preserve the condensable fractions.

Chemically, this tendency coincides with the faster secondary cracking reactions which occur at higher temperatures. At 500 °C, the main fragments from polypropylene break apart more often, leading to shorter hydrocarbons that stay in the gas phase because of their low molecular weight. Under these conditions, the product mix depends not only on the initial catalytic break of the polymer chain but also on the instability of the intermediate vapors within the reactor. This helps explain why AT-ZN produces much more gas, since its catalytic surface does not seem to guide the reaction toward stable liquid products. H-ZN performs better, likely because its protonic nature still supports more controlled cracking, which slows down the change from liquid intermediates to permanent gases [41].

### 3.3. GC-MS Analysis

To determine the effect of catalyst change and operating conditions on the liquid fraction, pyrolysis oils were analyzed by GC–MS, categorized from carbon-number ranges corresponding to fuel cuts. They focused on light gasoline (C6–C11), diesel-range hydrocarbons (C12–C20), light waxes or kerosene-like fractions (C21–C28), and heavy hydrocarbons (C29–C40), as they showed cracking extent and catalyst selectivity. The adjusted zeolite composition, determined from product yield, altered both the quantity and composition of the recovered liquid. The acidic catalyst H-ZN was more favorable in the present experiment, and especially at medium-to-high temperatures, the diesel fraction significantly increased. In comparison, AT-ZN provided more light hydrocarbons at elevated temperatures, whereas AA-ZN exhibited intermediate results. The GC–MS results indicate that catalyst acidity, as well as the operating conditions, can affect whether vapors from polypropylene are stabilized as diesel-range products or broken down into lighter fractions.

**Figure 6:**
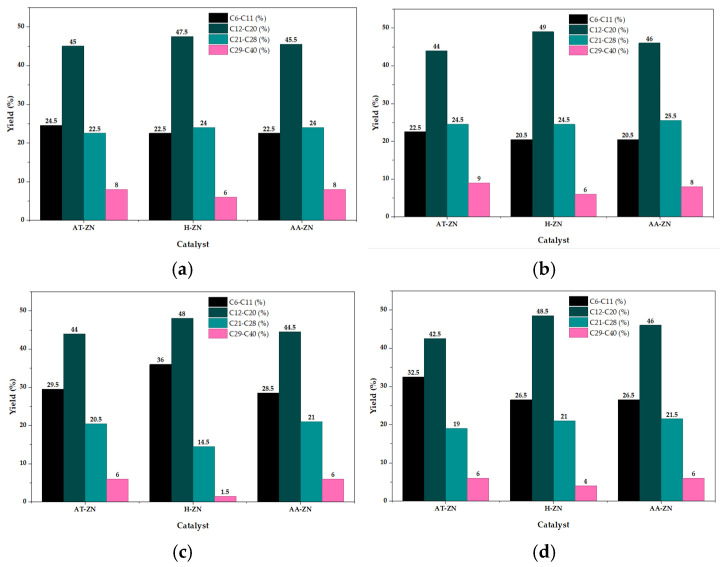
Distribution of hydrocarbon fractions in the liquid products obtained from the catalytic pyrolysis of polypropylene at 400 °C under different operating conditions: (**a**) 5 wt% catalyst and 10 °C min^−1^, (**b**) 5 wt% catalyst and 20 °C min^−1^, (**c**) 10 wt% catalyst and 10 °C min^−1^, and (**d**) 10 wt% catalyst and 20 °C min^−1^.

As depicted in Figure 6a–d, at 400 °C, all the catalysts generate liquid products, mostly in the diesel-range fractions (C12–C20). This indicates that mild thermal conditions are more beneficial for condensable hydrocarbons than for excessive cracking to lighter compounds. H-ZN achieves the best selectivity for C12–C20 in this range, exhibiting 47.5% (6a), 49.0% (6b), 48.0% (6c), and 48.5% (6d), which are better than those of AT-ZN and AA-ZN. These results suggested that the acidic type of zeolite provides an optimal balance between cracking polymer chains and stabilizing fragments, and consequently produces higher quantities of hydrocarbons in the diesel range. The acid sites of H-ZN are tailored to moderate β-scission of polypropylene chains, thus avoiding too much production of either light gasoline or heavy waxes. The low C29–C40 fraction in H-ZN (1.5% in Figure 6c) supports this observation, demonstrating that heavy residues are strongly suppressed under these conditions [6,37].

**Figure 7:**
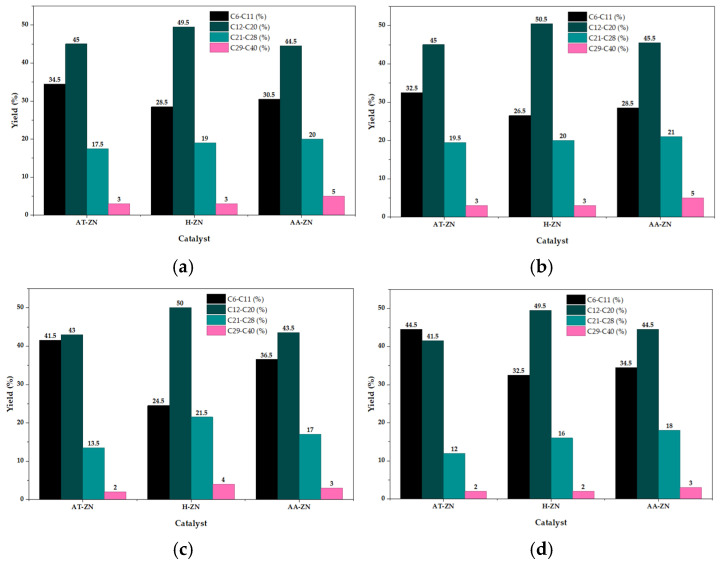
Distribution of hydrocarbon fractions in the liquid products obtained from the catalytic pyrolysis of polypropylene at 450 °C under different operating conditions: (**a**) 5 wt% catalyst and 10 °C min^−1^, (**b**) 5 wt% catalyst and 20 °C min^−1^, (**c**) 10 wt% catalyst and 10 °C min^−1^, and (**d**) 10 wt% catalyst and 20 °C min^−1^.

The opposite case yields a broader range of products and low molecular selectivity. AT-ZN preferred C6–C11 by increasing the catalyst loading; the light fraction increased to 32.5% (Figure 7d) from 24.5% (Figure 7a), and the diesel fraction decreased from 45.0% to 42.5%. The ability of thermal activation, in and of itself, to produce a catalytic surface that does not provide the same stabilizing advantage for intermediate hydrocarbons reflects a significantly increased cracking toward gasoline-range products. AA-ZN showed intermediate behavior with stable C12–C20 yields (44.5–46.0%) and intermediate C21–C28 fractions (21.0–25.5%), indicating a more balanced but less selective cracking environment than H-ZN. Similarly, as shown in Figure 7c, the increase in C6–C11 (36.0%) increases with the H-ZN concentration at 10 wt% and 10 °C min^−1^, whereas the decrease is sharp in C21–C28 (14.5%) and C29–C40 (1.5%). This phenomenon suggests that the secondary cracking of heavier intermediates is active even at 400 °C, a fact supported by the fact that highly concentrated active sites drive the product toward lighter molecules. However, under Figure 7d, a faster heating rate partly compensates for this effect, restoring the dominance of the diesel fraction in H-ZN (48.5%) and moderating the excessive growth of the light fraction [38].

As shown in Figure 7a–d, at 450 °C, the product distribution remains centered in the diesel fraction (C12–C20), but the effect of temperature becomes more evident through a progressive redistribution toward lighter hydrocarbons, particularly AT–ZN. Under all four operating conditions, H-ZN maintains the highest selectivity toward C12–C20, reaching 49.5% (7a), 50.5% (7b), 50.0% (7c), and 49.5% (7d), which confirms that the acidic form preserves the best capacity to direct polypropylene cracking toward the diesel range even under stronger thermal input. From a chemical standpoint, this behavior indicates that H-ZN provides an acidity level that promotes effective chain scission while limiting excessive fragmentation of condensable intermediates. In parallel, the C29–C40 fraction remains very low for this catalyst (2–4%), which indicates efficient suppression of heavy wax-like products and a narrower, more desirable hydrocarbon distribution [38,42].

**Figure 8:**
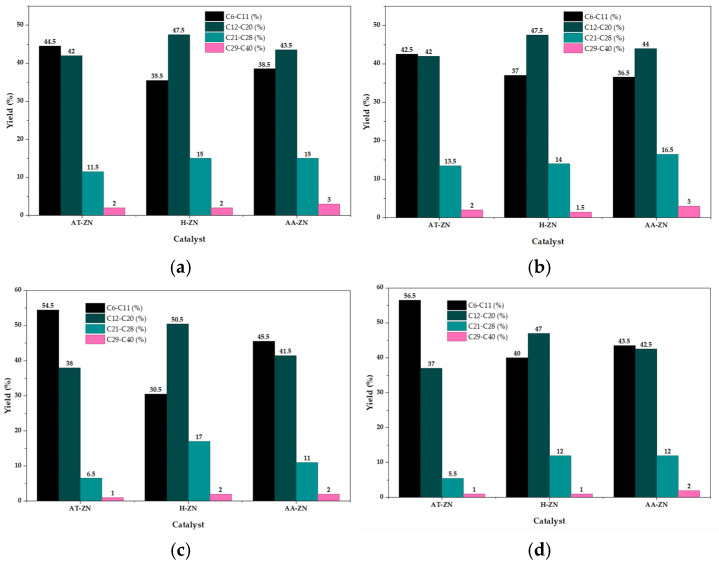
Distribution of hydrocarbon fractions in the liquid products obtained from the catalytic pyrolysis of polypropylene at 500 °C under different operating conditions: (**a**) 5 wt% catalyst and 10 °C min^−1^, (**b**) 5 wt% catalyst and 20 °C min^−1^, (**c**) 10 wt% catalyst and 10 °C min^−1^, and (**d**) 10 wt% catalyst and 20 °C min^−1^.

AT-ZN behaves differently with respect to temperature changes. The C6–C11 fraction rises from 32.5–34.5% (in Figure 7a,b) to 41.5–44.5% (in Figure 7c,d), and the diesel fraction decreases from 45.0% to 43.0–41.5%. In this way, at 450 °C, the zeolite catalyst was able to easily convert mid-range hydrocarbons into gasoline-range molecules. This signifies that a more vigorous depolymerization stage has begun, wherein the unstable polypropylene intermediates are more easily broken apart. AA-ZN exhibits an intermediate response in C12–C20 ratios of 43.5% to 45.5%, and moderate C6–C11 fractions (28.5–36.5%), suggesting that it is less selective but remains stable in a cracking environment. As noted in Figure 8d, AT-ZN reaches the highest C6–C11 value of 44.5%, compared with the diesel fraction at 41.5%; thus, increased catalyst loading and faster heating lead to the production of lighter fuel. Overall, Figure 7a–d shows that 450 °C is a transitional regime: H-ZN continues to favor diesel-range products, while AT-ZN shifts toward gasoline-rich liquids as cracking severity increases [39,40].

From Figure 8a–d, the chemical orientation of pyrolysis oil shifts more toward lighter hydrocarbons, and the resulting chemical structure indicates that the system operates in a high-severity cracking regime at 500 °C. AT-ZN results in the most significant increase in gasoline-range fraction C6 to C11, which rises from 44.5% in Figure 8a to 56.5% in Figure 8d. The diesel fraction (C12–C20) similarly decreases between 42.0% and 37.0%. This trend indicates that increasing temperatures and heated zeolite cause less selective activity in the medium, resulting in additional degradation of diesel-range intermediates into lighter molecular fractions, which is to say that secondary cracking can be more intense, medium-chain hydrocarbons become less stable, and more of them separate. Put simply, a larger share of compounds is added to the gasoline pool. More precisely, the reduction in the C21–C28 and C29–C40 fractions, especially in Figure 8c,d, suggests that heavier intermediates are being converted into lighter products [6].

In addition to the above, we have also selectively catalyzed the diesel fraction with H-ZN, exhibiting the highest selectivities among Figure 8a–d, which are 47.5% for Figure 8a,b, 50.5% for Figure 8c, and 47.0% for Figure 8d, confirming that the acidic phase is the highest to maintain the medium range of hydrocarbons at high thermal input. The presence of acid sites (in H-ZN) favors controlled, selective scission, thereby promoting C12–C20 product formation and maintaining high fragmentation. It shows intermediate behavior, between [41.5–44.0% C12–C20], and concurrently higher C6–C11 at higher catalyst loadings. Significantly, the C29–C40 fraction for each catalyst (1–3%) is negligible, indicating that heavy waxes remain highly suppressed at 500 °C, regardless of the catalytic agent. The general trends in Figure 8a–d demonstrate that the differences in catalysts are exacerbated at 500 °C: AT-ZN undergoes a transition toward hydrocarbons of a gasoline range, H-ZN offers the highest selectivity toward diesel, and AA-ZN remains intermediate. This confirms the effect of catalyst acidity on the nature of pyrolysis under extreme conditions that favor lighter fuels or that keep a diesel-centered product slate [36,37,42,43].

From a process-evaluation standpoint, the GC–MS analysis constitutes a key element of the present study, since the relevance of the liquid fraction is determined not only by its yield but also by its compositional proximity to fuel-relevant hydrocarbon domains. In this context, the predominance of the C12–C20 interval in the liquid products, particularly in the H-ZN system under mild-to-intermediate operating conditions, supports the interpretation that the process selectively enriched a diesel-range fraction rather than generating a fully undefined hydrocarbon mixture. This interpretation is consistent with previous reports on polypropylene-derived oils and related fuel studies, where compounds such as dodecane, tetradecane, pentadecane, hexadecane, octadecane, nonadecane, and alkyl-substituted naphthalene derivatives have been identified within the middle-distillate region. Likewise, pyrolysis-derived liquid fractions enriched in n-alkanes and 1-alkenes spanning the C12–C20 range have been described as compositionally close to diesel-like cuts. In the present work, this interpretation is further reinforced by the literature-based cross-reference presented in Table 2, which shows that a substantial number of compounds associated with the C12–C20 window have already been reported in previous polypropylene pyrolysis studies. Accordingly, although the GC–MS approach adopted here is based on carbon-number distribution rather than exhaustive peak-by-peak molecular identification, the results provide a chemically and technologically sound basis for discussing diesel-oriented selectivity in the liquid phase. Nevertheless, this assignment should be understood as a fuel-range classification and not as evidence of complete compositional equivalence to commercial diesel. A further limitation of the present study is the absence of gas-phase characterization. This aspect is relevant because previous investigations on polypropylene pyrolysis have shown that the gaseous products may contain hydrogen, methane, carbon monoxide, and carbon dioxide, indicating that CO_2_ should be considered as part of the overall product distribution. Consequently, future studies should incorporate gas-phase analysis in order to establish a more complete mass balance and to strengthen the environmental assessment of the process.

### 3.4. Correlation Matrix for the Variables of the Catalytic Pyrolysis Process of PP

To further resolve the relationships between operating variables and product distribution, Pearson correlation analysis was performed separately for each catalytic system. As shown in Figure 6, this approach makes it possible to identify catalyst-dependent linear associations between pyrolysis temperature, heating rate, catalyst loading, and the liquid, solid, and gaseous fractions, thereby providing a more integrated statistical view of how each modified zeolite governs the reaction network.

**Figure 9:**
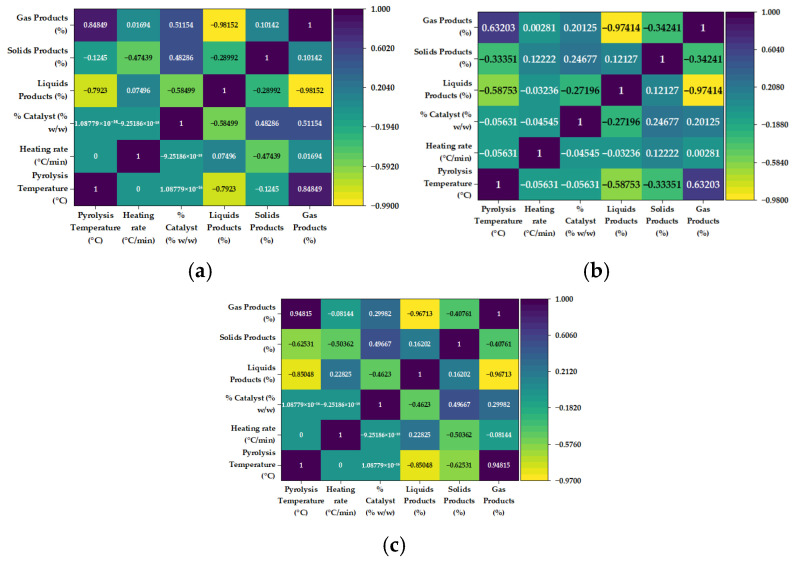
Heatmaps of the Pearson correlation matrices for the catalytic pyrolysis of polypropylene over modified natural zeolites: (**a**) AT-ZN, (**b**) H-ZN, and (**c**) AA-ZN. The color scale and the numerical values inside each cell represent the Pearson correlation coefficient (r) between the operating variables and the product fractions.

From a statistical perspective, each value in Figure 9a–c represents the strength and direction of the linear relationship between two variables within the experimental domain. Thus, coefficients close to +1 indicate that both variables increase together; coefficients close to −1 indicate that one variable increases as the other decreases, and coefficients near 0 indicate the absence of a relevant linear trend. For instance, a value such as 0.948 denotes a very strong positive association, whereas −0.981 denotes an almost perfectly inverse relationship. In practical terms, these coefficients do not imply causality on their own. Still, they do reveal which process variables are most strongly coupled to the redistribution of mass among liquid, solid, and gas products [48,49].

All three catalytic systems exhibit strong negative correlations between the liquid and gas yields, with respective coefficients of −0.9815 (Figure 9a) for AT-ZN, −0.9741 (Figure 6b) for H-ZN, and −0.9671 (Figure 6c) for AA-ZN. This is the strongest correlation in the dataset, indicating that liquid and gaseous fractions are negatively correlated across the operating range. From a physicochemical perspective, this exemplifies the competitive characteristics of polypropylene pyrolysis: conditions that promote the preservation and condensation of intermediate hydrocarbons improve liquid recovery, whereas those that increase bond cleavage tip the mass balance toward permanent gases [6]. In AT-ZN (Figure 6a), we observe a strong positive correlation between temperature and gas yield (r = 0.8485) and a strong negative correlation between temperature and liquid yield (r = −0.7923). So, this suggests that the catalyst is highly dependent on the thermal severity. As the temperature increases, the rate of secondary cracking increases, so that the more volatile intermediates of polypropylene are unlikely to be condensable hydrocarbons but rather become lighter gases. This idea is supported by the positive (r = 0.5115) and negative (r = −0.5850) relationships between catalyst loading and gas output. Here, more catalyst does not increase liquid selectivity; it only causes fragmentation. The correlation with heating rate is almost zero; for instance, temperature and catalyst amount primarily control product distribution for AT-ZN, not heating rate [50].

Regarding H-ZN (Figure 9b), when considering temperature, we find significant correlations with gas (r = 0.6320) and liquids (r = −0.5875). Still, these associations are less pronounced than in AT-ZN and AA-ZN. That suggests a more moderate response to thermal input. Statistically, the system is driven by a broader combination of variables, thus corresponding with the characteristics of a more selective catalyst. At a chemical level, since H-ZN is protonic, it yields a higher density of Brønsted acid sites, which ensures a controlled cracking mechanism and efficient stabilization of condensable intermediates. Weak correlations between heating rate, catalyst loading, and product yields also suggest that selectivity in this catalyst results from a balanced interaction between acidity and reaction conditions. On AA-ZN (Figure 9c), temperature shows the strongest positive correlation with gas production (r = 0.9482) and the strongest negative correlation with liquid yield (r = −0.8505), indicating that this system is highly sensitive to higher temperatures. As the temperature rises, the liquid fraction becomes less stable, causing it to convert into gas rapidly. The negative correlation between heating rate and solids (r = −0.5036) means that faster heating reduces the formation of solids. In contrast, the positive correlation between catalyst loading and solids (r = 0.4967) suggests that higher catalyst loading leads to greater residue or over-cracking. The differences in reactivity of the three zeolites when the process variables are considered are summarized from the correlations indicated in Figure 9a–c. AT-ZN and AA-ZN are more temperature-sensitive. Still, H-ZN exerts an indirect and selective effect, consistent with its potential to mediate the conversion of polypropylene into the liquid phase [6,50].

### 3.5. Analysis of Variance (ANOVA): AT-ZN’s “Brute Force” vs. H-ZN’s “Precision Synergy”

AT-ZN operates primarily as a single factor, with little to no cross-interactions occurring. With a thermally activated catalyst, the main determinants of yield are catalyst loading (F = 720.00) and pyrolysis temperature (F = 669.99). This shows that the catalytic surface of AT-ZN mainly promotes cracking with increased process severity, whereas any interplay among variables is limited. This behavior is similar to catalytic pyrolysis systems, which are thermally driven and, with higher catalyst dosage, promote greater fragmentation rather than selectivity. The significant catalyst-loading effect indicates that there is a need for an adequate effective surface for measured cracking in only thermally activated zeolite; the broader interaction network in more acidic zeolitic forms is not observed [50]. A distinct response towards H-ZN was clearly seen. Almost every interaction term was statistically significant at *p* < 0.05, suggesting that the acidic form arises from a combination of temperature, heating rate, and catalyst loading, rather than from each effect acting independently. This is consistent with a zeolitic system in which several Brønsted acid sites can bring polymer chains into the activation of carbocation-based intermediates, which may then be further broken, rearranged, or cracked depending on acidity and heating. A large temperature × heating rate interaction (*p* = 0.0034) and a three-factor interaction (*p* = 0.019) indicate that the catalytic environment H-ZN establishes is more temperature-sensitive. Higher acidity can even produce more liquid, though if operating conditions are out of balance, over-cracking is inevitable. This enhanced sensitivity is, to some extent, a justifiable reason why H-ZN can reach liquid values greater than 70% only over a limited range of operating conditions and is especially good for changing a reactor to produce more long-range diesel products. Overall, H-ZN becomes the best active catalyst of the three, with superior liquid recovery ability, greater variable interaction, and more controlled behavior, to obtain the most valuable hydrocarbons and minimize coke generation and process inefficiencies [32]. See Table 3 and Table 4.

### 3.6. Pearson Correlation Analysis of Carbon-Number Distribution in Pyrolysis Oils

To refine the interpretation of the GC–MS results, Pearson correlation matrices were constructed separately for AT-ZN, H-ZN, and AA-ZN by relating the operating variables to the relative abundance of the main carbon-number fractions in the liquid products. This analysis allows identification of the dominant variables governing molecular redistribution within the oil phase. It clarifies whether each catalyst tends to preserve the diesel-range fraction (C12–C20) or promote further cracking toward lighter hydrocarbons.

**Figure 10:**
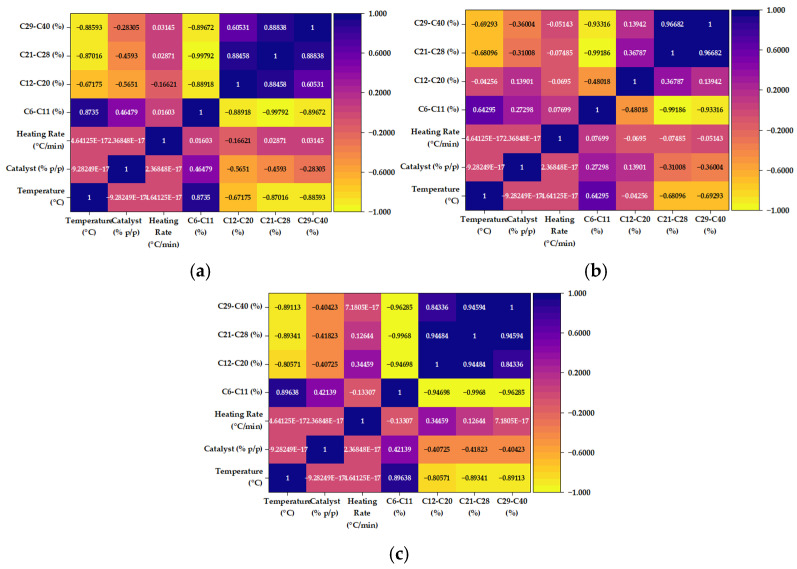
Heatmaps of Pearson correlation coefficients describing the relationships between operating variables (temperature, catalyst loading, and heating rate) and hydrocarbon fractions in the liquid products obtained from catalytic pyrolysis of polypropylene using (**a**) AT-ZN, (**b**) H-ZN, and (**c**) AA-ZN.

Temperature mainly controls the distribution of hydrocarbons across AT-ZN. Higher temperatures enhance C6–C11 (r = 0.8735) and diminish C12–C20 (r = −0.6718), C21–C28 (r = −0.8702), and C29–C40 (r = −0.8859). As a consequence, the more severe the reaction, the more the liquid product shifts towards lighter hydrocarbons, reducing diesel and heavier fractions. Catalyst loading has a similar but weaker effect, and heating rate has little impact. In H-ZN (Figure 10b), temperature still favors C6–C11 (r = 0.6430) and reduces C21–C28 (r = −0.6810) and C29–C40 (r = −0.6929), but its correlation with C12–C20 is nearly negligible (r = −0.0426). These findings illustrate that H-ZN can selectively inhibit heavier hydrocarbons and retain the diesel-range fraction, indicating enhanced compositional stability. In contrast, AA-ZN (Figure 10c) has a more significant temperature-dependent mechanism that shows a positive trend of correlation for C6–C11 (r = 0.8964) and a negative one for C12–C20 (r = −0.8057), C21–C28 (r = −0.8934), and C29–C40 (r = −0.8911). This tendency reflects the greater tendency of lighter compounds to shift toward lighter compounds under the aforementioned extreme conditions. Overall, the over-cracking tendency of AT-ZN and AA-ZN is more common and is also expressed in Figure 10a–c. In contrast, H-ZN stabilizes the diesel-range fraction more effectively within this operational window [48,49].

## 4. Conclusions

This study shows that the nature of natural zeolites is significantly altered, as described, which significantly impacts the efficiency and selectivity of polypropylene catalytic pyrolysis. If the catalyst was modified, the conversion rate increased, as did the thermal cracking range, product phase distribution, and carbon-number profile of the liquid fraction. Of the materials tested, H-ZN exhibited the greatest liquid yields and the most consistent enrichment of the diesel-range fraction (C12–C20). The protonic form gives a better balance between the acidity and selectivity for controlling chain scission, limiting excess gas formation, and preventing heavy wax-like products from remaining. We observed increased sensitivity to reaction severity in AT-ZN and a greater preference for light gasoline-range hydrocarbons (C6–C11), whereas AA-ZN exhibited intermediate behavior. Zeolite manipulation not only changes conversion but also controls the distribution of polypropylene-based intermediates among light, middle, and heavy hydrocarbon fractions. Statistical analyses also confirmed the process—no single variable can explain the process. Part of the selectivity also depends on temperature, catalyst loading, heating rate, and catalyst type. At moderate conditions—particularly around 400 °C with reduced catalyst loading—liquid recovery was highest, and the product profile remained diesel-focused. Conversely, secondary cracking is easier at high temperatures, and the product distribution will be biased toward lighter hydrocarbons or gases. These results suggest that modified natural zeolites are efficient, affordable, and scalable catalysts for the recycling of polypropylene waste. H-ZN is better suited for converting polypropylene into diesel, while AT-ZN is better suited for lighter gasoline-range products. The paper details and provides practical guidance on tuning product selectivity in catalytic plastic pyrolysis, thereby supporting the development of low-cost, circular technologies for waste-to-fuel conversion.

## Figures and Tables

**Table 1:** Typical physicochemical properties of natural and modified zeolites used in catalytic pyrolysis.

Catalyst	Si/Al Ratio	BET Surface Área (m^2^/g)	Pore Volume (cm^3^/g)	Average Pore Diameter (nm)	Acidity (mmol NH_3_/g)	Acid Site Type	Crystallinity (XRD, %)
Natural ZN	4.7–4.9	25–26	0.08–0.09	4–5	0.16–0.18	Weak Lewis	99–100
AT-ZN (Thermal)	4.9–5.1	36–37	0.10–0.11	6–7	0.23–0.25	Lewis dominant	92–93
AA-ZN (Acid-treated)	6.9–7.1	89–90	0.18–0.19	7–8	0.46–0.51	Lewis + moderate Brønsted	79–80
H-ZN (Acid form)	7.9–8.0	133–134	0.28–0.32	8–9	0.71–0.72	Strong Brønsted dominant	80–82

**Table 2:** Literature-based cross-reference of representative compounds associated with the diesel-range fraction (C12–C20) in polypropylene pyrolysis oils and related fuel studies.

Compound	Mangesh et al. (2019) [44]	Kalargaris et al. (2017) [45]	Melder et al. (2023) [46]	Quesada et al. (2020) [47]	Present Work
2,4,6-Trimethyl-1-nonene					+
2,4,6,8-Tetramethyl-1-nonene					+
2,4,6,8-Tetramethyl-1,6-nonadiene					+
2,4,6,8-Tetramethyl-1-undecene					+
Decenetrimethyl	+				+
Dodecene	+				+
Tridecene	+				+
Tetradecene	+				+
Pentadecene					+
Hexadecene					+
Heptadecene					+
Octadecene					+
Nonadecene					+
Eicosene					+
9-Eicosene (E)				+	+
Dodecane	+		+	+	
Tetradecane	+			+	
Pentadecane	+			+	+
Hexadecane	+			+	
Octadecane	+			+	
Nonadecane	+			+	
Dodecane, trimethyl	+				+
Dodecylbenzene					+
Tridecylbenzene					+
Tetradecylbenzene			+		+
Pentadecylbenzene					+
Hexadecylbenzene					+
Heptadecylbenzene					+
Octadecylbenzene					+
Nonadecylbenzene					+
Eicosylbenzene					+
Naphthalene	+	+			+
Methylnaphthalene	+				+
Dimethylnaphthalene	+				+
Trimethylnaphthalene	+				+
Ethylnaphthalene					+
Phenanthrene					+
2-Phenylnaphthalene					+
Benzene	+	+	+		
Toluene		+	+	+	
Ethylbenzene		+	+	+	
Xylene		+	+		
Benzene, diethyl	+				
Benzene, dimethyl	+				
Benzene, trimethyl	+				
Phenol		+			
Undecane, 4,6-dimethyl-				+	
2-Isopropyl-5-methyl-1-heptanol				+	
2-Methyl-7-octadecyne				+	+
2-Methyltetracosane	+				
Cyclotetradecane, 1,7,11-trimethyl-4-(1-methylethyl)-				+	
Dodecane, 1-cyclopentyl-4-(3-cyclopentylpropyl)-				+	
1-Hexadecanol				+	
11-Dodecen-1-ol, 2,4,6-trimethyl-, (R, R, R)-				+	

**Table 3:** Analysis of variance (ANOVA) of the yield of products in the pyrolysis of PP with AT-ZN catalyst. The asterisk (*) indicates the corresponding highlighted values/conditions in the table; it does not denote multiplication. The same interpretation applies to all identical highlights.

	Degrees of Freedom	Sum of Squares	Mean Square	F-Value	*p*-Value
Pyrolysis temperature	2	399.76333	199.88167	669.99441	<0.0001
Heating rate	1	3.52667	3.52667	11.82123	0.00491
Amount of catalyst	1	214.80167	214.80167	720.00559	<0.0001
Pyrolysis temperature * Heating rate	2	0.00333	0.00167	0.00559	0.99443
Pyrolysis temperature * Amount of catalyst	2	0.00333	0.00167	0.00559	0.99443
Heating rate * Amount of catalyst	1	6	6	20.11173	7.46 × 10^−4^
Pyrolysis temperature * Heating rate * Amount of catalyst	2	0	0	0	1
Model	11	624.09833	56.73621	190.17725	<0.0001
Error	12	3.58	0.29833		
Total Corrected	23	627.67833			

**Table 4:** Analysis of variance (ANOVA) of the yield of products in the pyrolysis of PP with H-ZN catalyst. The asterisk (*) indicates the corresponding highlighted values/conditions in the table; it does not denote multiplication. The same interpretation applies to all identical highlights.

	Degrees of Freedom	Sum of Squares	Mean Square	F-Value	*p*-Value
Pyrolysis temperature	2	163.50526	81.75263	29.85451	<0.0001
Heating rate	1	4.152	4.152	1.51623	0.24387
Amount of catalyst	1	41.35323	41.35323	15.10142	0.00254
Pyrolysis temperature * Heating rate	2	54.26129	27.13065	9.9076	0.00346
Pyrolysis temperature * Amount of catalyst	2	51.89051	25.94525	9.47471	0.00405
Heating rate * Amount of catalyst	1	15.70939	15.70939	5.73677	0.03554
Pyrolysis temperature * Heating rate * Amount of catalyst	2	31.7339	15.86695	5.79431	0.01911
Model	11	350.60352	31.87305	11.63943	0.00154
Error	11	30.12205	2.73837		
Total Corrected	22	380.72557			

## Data Availability

The original contributions presented in this study are included in the article. Further inquiries can be directed to the corresponding author.
